# Effect of Graphene Nanosheet Addition on the Wettability and Mechanical Properties of Sn-20Bi-xGNS/Cu Solder Joints

**DOI:** 10.3390/ma13183968

**Published:** 2020-09-08

**Authors:** Wenchao Yang, Zuojun Yang, Yaokun Fu, Aihua Yu, Junli Feng, Yongzhong Zhan

**Affiliations:** 1 School of Materials Science and Engineering, South China University of Technology, Guangzhou 510641, China; ywch053@163.com; 2 Guangxi Key Laboratory of Processing for Non-ferrous Metals and Featured Materials, School of Resources, Environment and Materials, Guangxi University, Nanning 530004, China; ykfu1117@163.com (Y.F.); aihua828118@yeah.net (A.Y.); 3 Shenzhen Customs Industrial Products Inspection Technology Center, Shenzhen 518067, China; yangzuojun2009@163.com (Z.Y.); fjlhhp@foxmail.com (J.F.); 4 Shenzhen Academy of Inspection and Quarantine, Shenzhen 518010, China

**Keywords:** lead-free solder, Sn-20Bi, graphene nanosheets, wettability, intermetallic compounds, hardness

## Abstract

Graphene nanosheets (GNSs) have an extensive application in materials modification. In this study, the effects of graphene nanosheets on the wettability of Sn-20Bi lead-free solder on copper (Cu) substrate and the growth behavior of intermetallic compound (IMC) layers at Sn-20Bi-xGNS/Cu solder joints were investigated. The experimental results indicate that the wettability of Sn-20Bi solder firstly diminished and then increased by the addition of GNSs. Meanwhile, a prism-shaped and scallop-shaped Cu6Sn5 IMC layer was clearly observed at the interface of the solder/substrate system. Moreover, it was found that a small amount of GNS addition can significantly inhibit the growth of the IMC layer at the interface as well as refine the microstructure. Additionally, by nano-indentation apparatus, it can be concluded that the hardness and elastic module of IMCs show the same variation trend, which firstly decreased and then increased. Besides, to intuitively demonstrate the reliability of IMCs, the relationship between the hardness and elastic module was established, and the ratio of hardness/elastic module (H/E) was adopted to characterize the reliability of IMCs. The results show that when the addition of GNSs was 0.02 wt%, the value of H/E is the minimum and the solder joint has the highest reliability.

## 1. Introduction

In the microelectronic packaging industries, traditional Sn-Pb solder alloys were widely used due to the low melting temperature, low cost and high mechanical properties [1]. However, due to the toxicity of lead, the environment and health concerns, the use of traditional Sn-Pb solder was forbidden. Therefore, the modern microelectronic packaging industries are paying attention to environmentally friendly electronic interconnecting materials. In recent years, the research work on lead-free Sn-based solders has been carried out extensively. Composite solder, such as Sn-Bi [2], Sn-Ag [3], Sn-Cu [4,5], Sn-Ag-Cu [6] and Sn-Zn [7], etc., are employed to improve the reliability of solder joints to extend the service life of electronic products. Among them, the Sn-Bi alloy is considered a promising alternative to lead-free solder for the interconnection of joints due to its low melting temperature, which can significantly reduce thermal impact in packaging processes. Besides, the cost of Bi is cheaper, which means it could be used for mass production in the electronics industry.

To date, many studies have been published about the wettability, microstructural development and mechanical properties of Sn-Bi solder alloy. Chen et al. [8] found that the effect of In addition on the wettability of solder. The results show that the wettability of solder improves with the addition of In, and the wettability is the best when the addition amount is 4 wt%, and then it decreased with further addition because Cu-Sn-In intermetallic compound (IMC) formed at the interface. Gain and Zhang [9] have found that nano Ni particles can decrease the contact angle of solder due to the formation of finely distributed Ni3Sn4 at the interface. Dong et al. [10] found that rare earth elements can improve the wettability of solder better than Ag. In addition, many studies show that nanoparticle addition to the solder has an active effect on the properties of Sn-Bi alloy solder joints, such as Mo [11], Y2O3 [12], BaTiO3 [13] and graphite [14]. However, the near eutectic alloy of Sn-58Bi has an inherent defect because of a high content of Bi, and the reliability of Sn-58Bi solder joints will deteriorate due to the Bi segregation during the service process, which can result in an increase in the brittleness of solder joints and a decrease in ductility. Thus, the Sn-20Bi solder alloy is developed to reduce the influence of the brittle Bi-rich phase. The results reported by Lai and Ye [15] show that the microstructure of Sn-20Bi solder is constituted by Bi particles and β-Sn phase and the Sn-20Bi solder alloy has a higher tensile strength than Sn-xBi (x = 10, 25, 35) solder alloys. Hence, the Sn-20Bi solder joint has a great performance in the interconnection of electronic packaging. However, with increasing packaging density and thermal stress in three-dimensional packaging, there is a higher requirement for Sn-20 Bi solder alloy. In our previous study [16], we investigated the microstructure and properties of Sn-20Bi-xAl (x = 0, 0.1, 0.3, 0.5 wt%) solder alloys. The results reveal that the addition of Al deteriorated the wettability and improved the hardness and the corrosion resistance of the Sn-20Bi alloys. To explore more possibilities of the applications of lead-free solder alloy, graphene nanosheets (GNSs) were introduced into Sn-20Bi solder. Graphene nanosheets proved to be effective for the reinforcement of the properties of solder, such as Sn-58Bi [17] and Sn-Ag-Cu [18]. However, the further study of Sn-20Bi solder alloy is still needed.

In this study, to further improve the mechanical properties of solder joint for the service reliability in severe circumstances, graphene nanosheets were incorporated into Sn-20Bi solder to improve the properties of the composite solder. Because of the frangibility of Sn-20Bi alloys, composite material technology was adopted to improve the homogeneity of various ingredients in the composite solder. After the reflow of composite solders on the Cu substrate, the wettability of Sn-20Bi-xGNS composite solders on the substrate were obtained and analyzed. Besides, the microstructure at the interface of Sn-20Bi-xGNS/Cu solder joints was investigated and the mechanical properties of IMCs at the interface were also determined.

## 2. Experiment Procedures

### 2.1. Materials

The composite solders of Sn-20Bi-xGNSs (x = 0, 0.02, 0.04, 0.06, 0.08, 0.1) were synthesized successfully using 99.5% Sn, 99.9% Bi and 99.5% GNSs by the powder metallurgy method. First, the Sn, Bi and GNS powders (each 90 g) were mixed for 300 min in a planetary ball mill with a speed of 150 r/min. Then, the milled powder was slowly put into a graphite mold with an inner diameter of 35 mm, and then cold pressed by a powder tablet press. The pressure was 7–8 MPa. The deformed powder was put into a vacuum hot-pressing sintering furnace together with the mold for hot-pressing sintering. The specific process parameters of sintering were as follows: sintering temperature was about 160 °C, heating rate was controlled at 5 °C/min, sintering pressure was 30 MPa, and holding time was 120 min.

### 2.2. Wettability

In order to prepare the wettability samples, cylindric composite solders with Ф 6.25 × 1.24 mm were cut from the sintered specimens. The flux was prepared with pure ZnCl_2_ and NH_4_Cl reagent according to the mass ratio of H_2_O:ZnCl_2_:NH_4_Cl of 50:45:5. A copper plate with 40 × 40 × 2 mm was ground with sandpaper and polished by fabric, then cleaned with an ultrasonic wave in acetone solution to remove oil stains. After that, the pure Cu plates were washed with deionized water, followed by soaking in 10% hydrochloric acid solution for 10 s to remove surface oxide, which was then removed with 3% sodium hydroxide solution and finally cleaned with deionized water and dried in cold air. To figure out the wettability of the solder, the solder blocks were placed in polished Cu substrate and were heated in a chamber electric furnace at 250 ˚C for 20 min. Spreading area and spreading ratio were used to measure the wettability of Sn-20Bi-xGNS composite solders. Spreading area was calculated by Adobe Photoshop, and the spreading ratio was expressed as:

(1) $$S=\frac{D-h}{D}\times 100\%$$

where *S* stands for the spreading ratio, *D* is the equivalent diameter of composite solder and *h* is the maximum height of solder alloy spreading on the surface of the copper substrate. The higher the spreading ratio is, the better the wettability of the solder is. Every final sample was tested five times and the average value was obtained.

### 2.3. Microstructural Analysis

Specimens were mounted in epoxy, then ground and polished. The microstructures were observed by scanning electron microscopy (SEM, Hitachi, Tokyo, Japan) and the phase analyses were determined by X-ray diffraction (XRD, RigakuD/Max 2500V, Tokyo, Japan) operated at 40 kV and 200 mA.

### 2.4. IMC Layer Thickness

The microstructures of the composite solder and morphological characteristics of IMCs at the interface of the solder joint were observed and analyzed by a scanning electron microscope (SEM). With the aid of Image-Pro Plus software, the thickness of the IMC layer at the Sn-20Bi-xGNS/Cu interface was obtained by dividing the area of the IMC (*S*) by the interface length (*L*), as shown in the following equation:

(2) $$T=\frac{S}{L}$$

where *T* is the equivalent thickness of the IMC layer. The thickness of IMC was obtained for more than five locations in the joint. The error bars were plotted by using the standard deviation of the measured data.

### 2.5. Hardness

The hardness and elastic modulus of IMCs at the solder joints were measured by nano-indentation apparatus. To figure out the effect of GNS content on the hardness and elastic modulus of IMCs at the Sn-20Bi-xGNS/Cu interface, the nano-indentation apparatus was applied, with a load of 20 mN, a load and unload rate of 100 mN/min, and dwelling for 5 s at maximum value. To ensure the accuracy of the hardness and elastic module of IMCs, at least 12 points were detected for each composite solder joint to obtain the average value. Besides, the ratio of hardness/elastic module (H/E) of IMCs at the solder joint was adopted to estimate the reliability of the solder joint.

## 3. Results and Discussion

### 3.1. Wettability of Sn-20Bi-xGNS Solder on Cu Substrate

**Figure 1:**
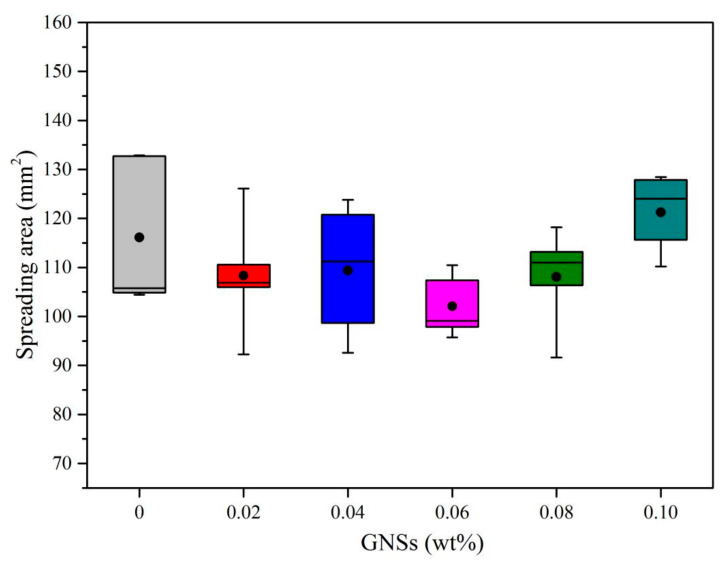
Spreading area of Sn-20Bi-xGNS solder on Cu substrate.

**Figure 2:**
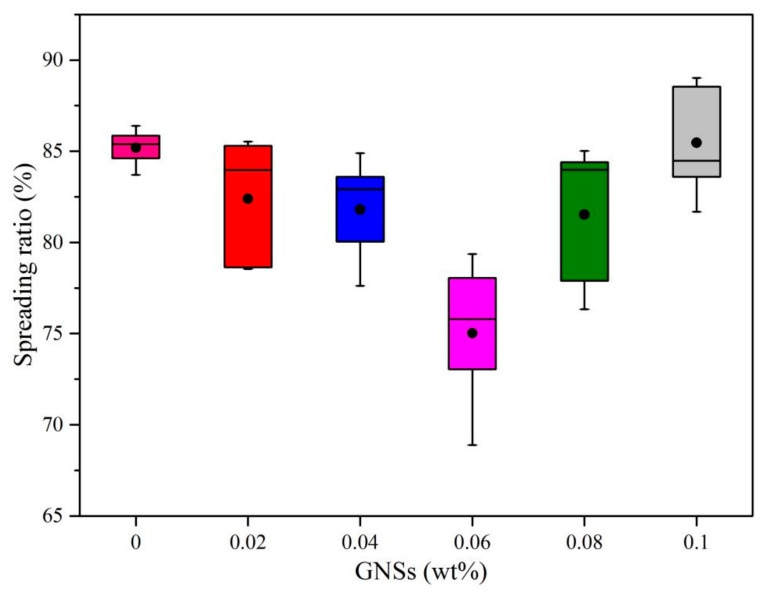
Spreading ratio of Sn-20Bi-xGNS solder on Cu substrate.

The wettability is an important prerequisite to form a good bonding between the solder and substrate. Usually, the wettability can be characterized by spreading area and spreading ratio, and the higher of spreading ratio, the better the bonding reliability [19,20,21]. Table 1 and Figure 1 show the spreading area of Sn-20Bi-xGNS solder on Cu substrate. Table 2 and Figure 2 show the spreading ratio of Sn-20Bi-xGNS solder on Cu substrate at various GNS contents. It was confirmed that both spreading area and spreading ratio were decreased with GNS addition until 0.06 wt% GNSs. Afterward, the wettability of the composite solder gradually increased with increasing addition of GNSs to exceed the wettability of the Sn-20Bi alloy. Based on the above results, it can be concluded that the Sn-20Bi-0.06GNS solder has minimum spreading area and spreading ratio, which means worse wettability.

At present, the research on the mechanism of the wettability of GNSs at solder joints has not been finalized. Wu et al. [17] have studied the effect of GNSs on the wettability of Sn-58Bi solder alloy. The results found that the spreading ratio of Sn-58Bi solder first increases because the GNSs can reduce the interfacial tension by lowering the interfacial surface energy between the melting solder and Cu substrate. However, the spreading ratio decreases with excess GNSs added into the solder, which may be attributed to the fact that a higher content of GNSs can make them flow out from the melting solder. In addition, Yin et al. [18] have investigated the impact of GNSs on the microstructure, tensile strength and wettability of the Sn-0.3Ag-0.7Cu solder. The wettability test result shows that the addition of GNSs can improve the wettability of solder and that the contact angle of composite solder is reduced by approximately 29% when 0.01% of GNS is added. They also point out that the GNSs can induce the attraction between rosin and composite solder, which reduces the surface tension between them. Besides, there are many researchers investigating the influence of GNSs on the wettability and properties of solder alloy [22,23,24,25].

In this study, the effect of GNS content on the spreading area and spreading ratio of Sn-20Bi-xGNSs composite solder is discussed. However, unlike the previous relevant research, a different phenomenon was observed and a reasonable guess is proposed about the experimental results obtained in this experiment. After reflowing in the chamber electric furnace, the graphene precipitation was found at the interface of the solder/substrate joint. In the reflowing process, the graphene nanosheet precipitation at the interface may prevent liquid-state solder spreading on the Cu substrate, which reduces the flowability of composite solder in a molten state. This is because the graphene nanosheets have strong adsorption capacity. This means it can adsorb on the surface of intermetallic compound and prevent the Cu from reacting with the matrix of Sn. Thus, it can hinder the composite solder reactive wetting with the Cu substrate. As for the macroscopic phenomenon, the spreading area of the solder on the Cu substrate was reduced, which means the wettability of the composite solder was decreased. When GNS content is below 0.06 wt %, with the GNS addition, the amount of GNS precipitation increases. Therefore, the flow resistance of solder in the molten state increases gradually along with the increase in the content of GNSs, which causes the spreading ratio to decline.

When the GNS addition is between 0.06 wt% and 0.1 wt%, the content of graphene is too high to be expelled from the molten solder and then be exposed on the periphery of the solder. The GNSs in the periphery of the solder can lower the interfacial tension between the solder and substrate. When this kind of effect is stronger than the effect of the GNSs’ hinderance of the reactive wetting during the process of soldering, the spreading area and the spreading ratio increase with the increase in the content of GNSs. Therefore, the wettability of the Sn-20Bi-xGNS solder improves due to the decrease in the surface tension between the molten solder and substrate caused by GNSs.

### 3.2. Microstructure of Sn-20Bi-xGNS/Cu Solder Joint

**Figure 3:**
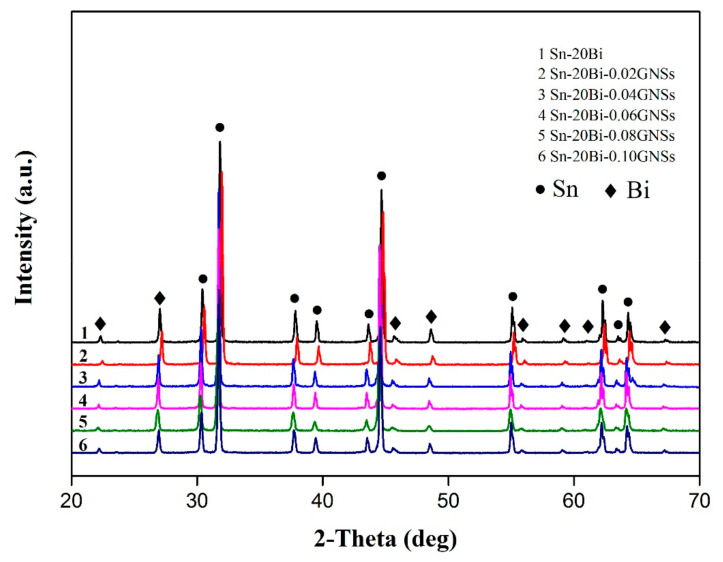
The XRD patterns of Sn-20Bi-xGNSs.

The microstructure of materials has a great effect on the mechanical properties of solder joints [26,27,28], thus, the microstructures of the Sn-20Bi-xGNS alloy and the IMCs at the Sn-20Bi-xGNS/Cu solder joint were studied. The XRD patterns of the solder alloys with different contents of GNSs are depicted in Figure 3. The XRD analysis of the Sn-20Bi solder shows that the two phases of Sn and Bi were detected. However, the XRD analysis of the composite solders with GNSs does not show the different diffraction patterns, and the new peak was not observed. This result indicates that GNSs did not react with Sn and Bi. Besides, the XRD analysis cannot reveal that the GNSs existed because the addition amount was extremely small.

**Figure 4:**
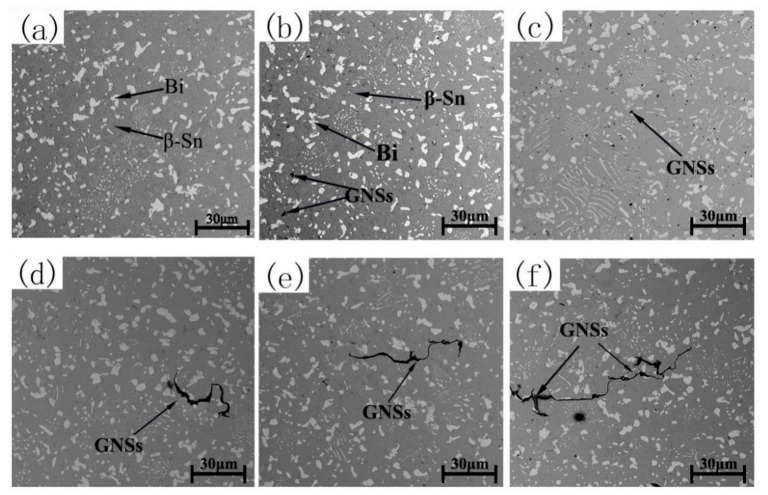
The SEM micrographs of Sn-20Bi-xGNSs, (**a**) x = 0, (**b**) x = 0.02, (**c**) x = 0.04, (**d**) x = 0.06, (**e**) x = 0.08, (**f**) x = 0.1.

**Figure 5:**
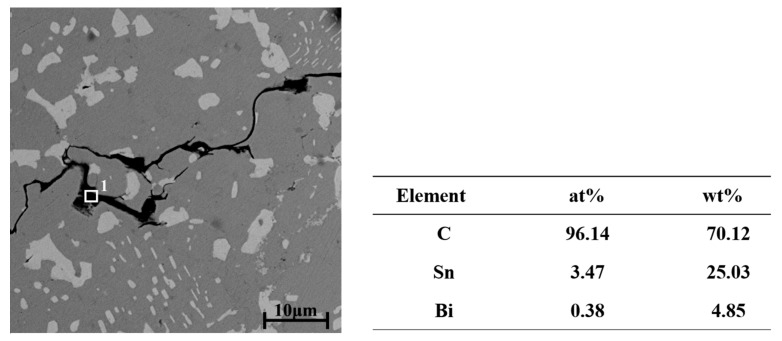
The energy spectrum analysis in Sn-20Bi-0.1GNSs.

To further understand the microstructure of the composite solder, the SEM micrographs of Sn-20Bi-xGNS solders are shown in Figure 4. In Figure 4a, only white Bi and gray β-Sn were observed in the solder matrix. With the addition of 0.02 wt% GNSs in the Sn-20Bi solder, black particles could be seen in the matrix. With the increasing addition of GNSs, the particles of GNSs increased in the solder, as shown in Figure 4b,c. However, when the addition of GNSs was up to 0.06 wt%, the number of black particles decreased, as shown in Figure 4d. However, a new black belt was observed in the matrix. This may result from the agglomeration of GNSs in the matrix due to the increasing amount. With the continued increase in GNSs, the black belt of GNSs also grew, as shown in Figure 4e,f. To definitively confirm the black particles and belt, the energy spectrum analysis was adopted. The local area in Figure 4f was chosen to be magnified in Figure 5, and the area with a white rectangle was analyzed. The result reveals that spectrum 1 is composed of 96.14 at% C, 3.47 at% Sn and 0.38 at% Bi. This means the black particles and black belt in the matrix are made up of GNSs.

**Figure 6:**
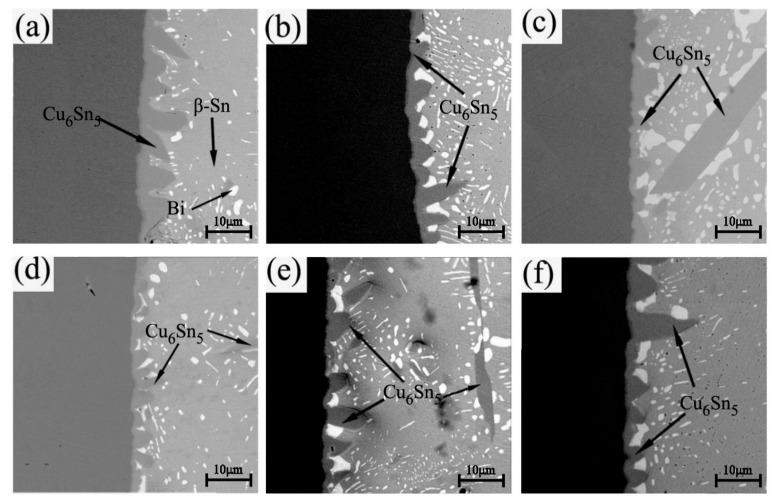
The interfacial microstructures at the Sn-20Bi-xGNS/Cu interface, (**a**) x = 0, (**b**) x = 0.02, (**c**) x = 0.04, (**d**) = 0.06, (**e**) = 0.08, (**f**) = 0.1.

Figure 6 shows the interfacial microstructures of the Sn-20Bi-xGNS/Cu solder joint reflowed at 250 ˚C for 20 min. After reflowing, the intermetallic compound layer was observed at the interface with prismatic and scallop shapes. From the comparison of the six figures in Figure 6, it can be found that when the GNS addition increased from 0 to 0.06 wt%, the number of prism shapes of IMCs declined, but the number of scallop shapes of IMCs increased and the single grain size decreased. When the content of graphene was in the range of 0.06–0.1 wt%, the prismatic IMCs increased again and the single grain size increased. Moreover, the IMC layer became discontinuous. However, the Sn-20Bi/Cu solder joint still had the biggest grain size and the most prismatic IMCs. This kind of change of grain shape may be due to the addition of GNSs, which had the effect of refining grains. In order to determine the composite of the IMCs, the dark gray part at the Sn-20Bi-0.02GNSs/Cu interface was analyzed by energy spectrum. According to the results of the energy spectrum analysis, the ratio of Cu atoms to Sn atoms is 6:5, which indicates that the dark gray compound with a scallop shape is Cu_6_Sn_5_. At the same time, Sona [5] and Xiong [6] also found that the Cu_6_Sn_5_ layer is formed at the interface between the Sn-based solder and Cu substrate.

### 3.3. Thickness of IMC Layer at Sn-20Bi-xGNS/Cu Interface

**Figure 7:**
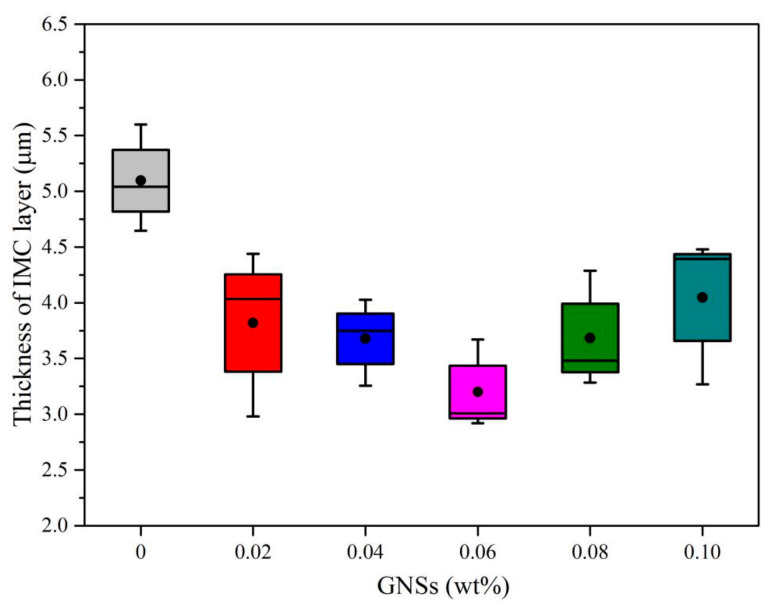
Thickness of IMC layer at Sn-20Bi-xGNS/Cu solder joint.

The formation of the IMC layer at the interface plays an important role in the reliability of microelectronic interconnections [29], and affects the service life of electronic products. The thicknesses of IMC layers at the Sn-20Bi-xGNS/Cu solder joint were measured. The obtained data are listed in Table 3 and plotted in Figure 7. Figure 7 shows that the interfacial IMC layer thickness significantly decreased with an increasing addition of GNSs until 0.06 wt%. After reflowing at 250 ˚C for 20 min, the thickness of IMCs at the Sn-20Bi/Cu interface is 4.99 µm. When the addition of GNSs increased up to 0.06 wt%, the thickness of the IMC layer at the Sn-20Bi-0.06GNS solder joint decreased to 3.2 µm, which is the thinnest IMC layer in this system. However, with increasing GNSs in the solder, the thickness of the IMC layer increases, as shown in Figure 7. The thickness of the IMC layer at the Sn-20Bi-0.1GNS/Cu interface is 4.05 µm, which is still thinner than that at the Sn-20Bi/Cu interface. As illustrated in Figure 7, when the addition of GNSs is 0.06%, GNSs have the greatest effect on the inhibition of growth of IMCs at the Sn-20Bi-xGNS/Cu solder joint.

Base on the above phenomenon, a reasonable assumption can be made. The GNSs have a positive effect on the inhibition of the growth of IMCs, which is important to the electronic product service process. During reflowing, the GNSs are absorbed at the interface of the solder/Cu. Because GNSs have a unique two-dimensional structure, this means that they have a larger specific surface area as well as powerful absorptivity, and can inhibit the atoms’ inter-diffusion at the solder/Cu interface. When the addition exceeds 0.06 wt%, the thickness of IMCs increases and the IMCs have obvious growth, as shown in Figure 6e,f. As mentioned in Figure 4d–f, GNSs appeared with a black belt shape because the excessive addition of GNSs caused agglomeration in the solder. In other words, the amount of GNSs absorbed at the solder/Cu interface was reduced due to agglomeration in the solder. Therefore, when the addition of GNSs exceeds 0.06 wt%, the effect of inhibition on the growth of IMCs also weakens, and the thickness of IMCs increases. Besides, although agglomeration occurred when the addition of GNSs was 0.06 wt%, there were still enough dispersed GNSs in the solder to inhibit the growth of IMCs. This means there is a value of addition between 0.04 and 0.06 wt% that has the best effect on the inhibition of inter-diffusion and no agglomeration.

### 3.4. The Mechanical Properties of IMCs

**Figure 8:**
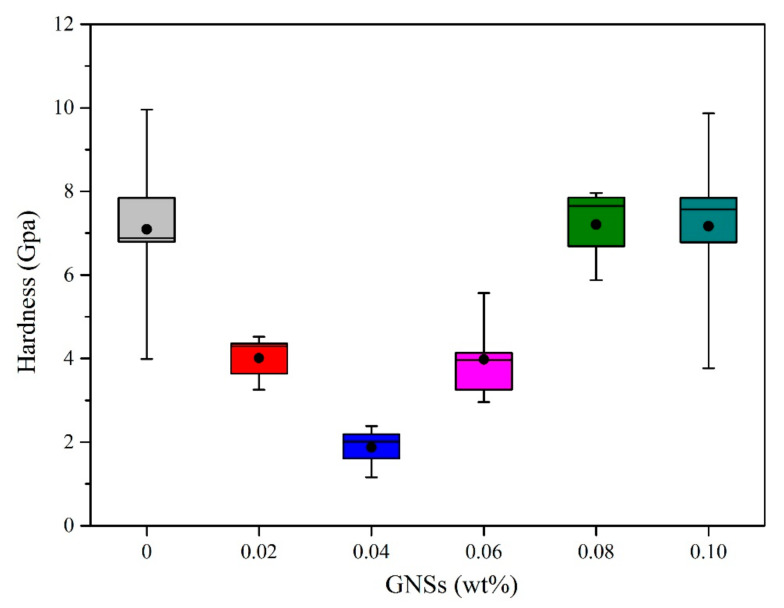
Hardness of IMCs at solder/Cu interface with various GNS contents.

**Figure 9:**
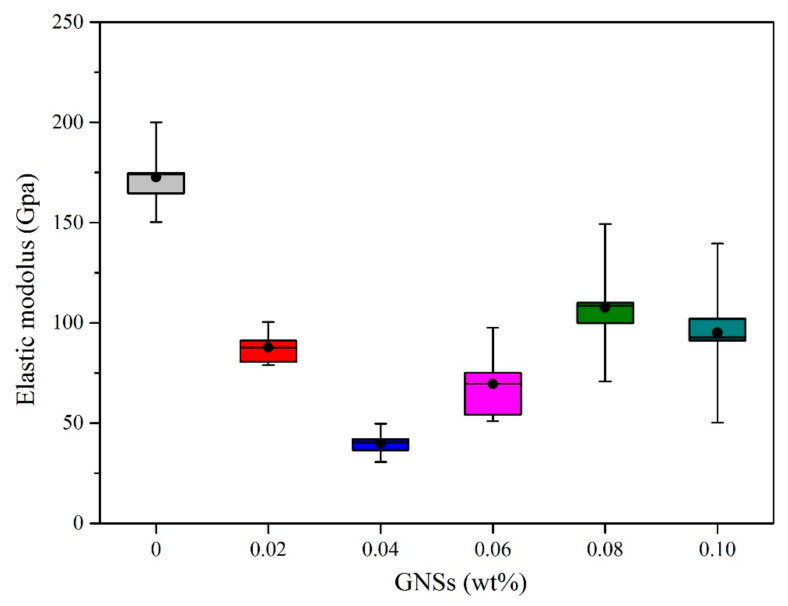
Elastic modulus of IMCs at solder/Cu interface with various GNS contents.

Intermetallic compounds, as an important component at interface between the solder and subtract, have a non-ignorable effect on the reliability of solder joints. In this study, the hardness and elastic modulus were adopted to characterize the mechanical properties of IMCs. Twelve points were tested in each sample to improve the accuracy of the mean value. The mean hardness and elastic modulus of IMCs between composite solders and the Cu substrate are listed in Table 4. Meanwhile, curve graphs of the hardness and elastic modulus of IMCs are shown in Figure 8 and Figure 9, respectively.

As is shown in Table 4, the initial hardness and elastic modulus of IMCs at the Sn-20Bi/Cu solder joint are 6.8841 and 174.7852 GPa. The variation tendency of hardness with GNS content is plotted in Figure 8. With increasing amounts of GNSs in the solder joint, the hardness of IMCs at the interface first decrease and then increase to 7.9628 GPa, and after that decrease to 6.7830 GPa, approximated initial status. Among the composite solder joints, the hardness has a minimum value at the Sn-20Bi-0.04GNS solder joint, and has a maximum value at the Sn-20Bi-0.08GNS solder joint. Generally, the grains of alloy are refined and the hardness of alloy increases as particles are added [30,31]. However, the hardness of IMCs in this study first decreased as GNSs were added, which still lacks an explanation. This will be further studied in future work.

To clearly understand the changes in the elastic modulus of IMCs with GNS content, the relationship between them is plotted in Figure 9. It can be concluded that the GNSs added in the solder could decrease the elastic modulus of IMCs. When the addition of GNSs is 0.04 wt%, the IMC has a minimum value of 36.4999 GPa. The elastic modulus of IMCs increased with increasing GNSs when the addition was more than 0.04 wt%, but reached a high peak at 0.08 wt%, and then decreased to 92.6174 GPa. It can be easily seen that the elastic modulus and hardness have the same variation trend as compared in Figure 8 and Figure 9. Kasimuthumaniyan [32] and Bao [33] have both pointed out that the hardness and elastic modulus of brittle material are correlated, as a higher value of hardness also means a higher value of elastic modulus. Similarly, in this study, the elastic modulus also shows the same variation trend with the change in hardness.

**Figure 10:**
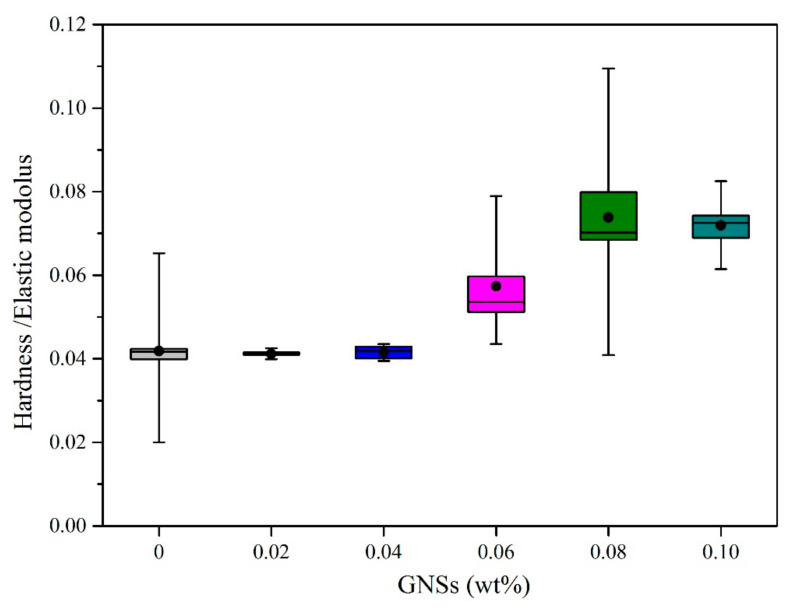
The ratio of hardness/elastic modulus of IMCs at the Sn-20Bi-xGNS/Cu composite solder joint.

In order to more intuitively demonstrate the reliability of IMCs, the value which can represent the relationship between reliability, hardness and elastic modulus needs to be established. In the past, the ratio of hardness versus elastic modulus (H/E) was applied to evaluate the reliability of hard brittle material. Bao [33] has proved that when the value of H/E decreases, the recovery resistance decreases and the energy dissipation increases. This means that when the value of H/E is smaller, elastic recovery is more difficult to achieve. In other words, the reliability of IMCs is worse. In this study, the ratio of hardness/elastic modulus (H/E) was adopted to characterize the reliability of IMCs. The ratio of H/E of IMCs at the Sn-20Bi-xGNS/Cu solder joint was analyzed and is listed in Table 5. The relationship between the ratio and content of GNSs is plotted in Figure 10. It can be concluded that the ratio of H/E first increases with the amount of GNSs added, and has a maximum value when the content of GNSs is 0.08 wt%. Therefore, the IMC has best elastic recovery and reliability when the GNS addition is 0.08 wt% in the composite solder joint.

## 4. Conclusions

The effects of graphene nanosheets on the wettability of Sn-20Bi solder and the mechanical properties of IMC layers at the Sn-20Bi-xGNS/Cu solder joint were investigated, and the main conclusions are as follows:

(1) Both spreading area and spreading ratio decrease with GNS addition until 0.06 wt%, which result from the adsorption of GNSs at the substrate surface, which lowers the reactive wetting. With increases in GNSs, the agglomeration of GNSs decreases the interfacial tension, thus, the Sn-20Bi-0.1GNS solder has the best wettability on the Cu substrate.
(2) When the content of GNSs exceeds 0.06 wt%, agglomeration happens at the composite solder. Besides, the addition of GNSs has the effect of refining Cu_6_Sn_5_ grains at composite solder joints.
(3) The hardness and elastic modulus of IMCs have the same variation trend. Furthermore, the IMC has best reliability when the GNS addition is 0.08 wt% at the composite solder joint.

## Figures and Tables

**Table 1:** Spreading area of Sn-20Bi solder with various content of graphene nanosheets (GNSs) on Cu substrate.

Solder	Spreading Area (mm^2^)
Sn-20Bi	116.13
Sn-20Bi-0.02GNSs	108.35
Sn-20Bi-0.04GNSs	109.40
Sn-20Bi-0.06GNSs	102.10
Sn-20Bi-0.08GNSs	108.07
Sn-20Bi-0.1GNSs	121.25

**Table 2:** Spreading ratio of Sn-20Bi-xGNS solder on Cu substrate.

Solder	Spreading Ratio (%)
Sn-20Bi	85.19
Sn-20Bi-0.02GNSs	82.40
Sn-20Bi-0.04GNSs	81.81
Sn-20Bi-0.06GNSs	75.03
Sn-20Bi-0.08GNSs	81.53
Sn-20Bi-0.1GNSs	85.46

**Table 3:** Thickness of intermetallic compound (IMC) layer at Sn-20Bi-xGNS/Cu solder joint.

Solder Joint	Thickness of IMC Layer (µm)
Sn-20Bi/Cu	4.99
Sn-20Bi-0.02GNS/Cu	3.82
Sn-20Bi-0.04GNS/Cu	3.68
Sn-20Bi-0.06GNS/Cu	3.20
Sn-20Bi-0.08GNS/Cu	3.68
Sn-20Bi-0.1GNS/Cu	4.05

**Table 4:** The hardness and elastic modulus of IMCs in Sn-20Bi-xGNS/Cu.

Solder Joints	Hardness (GPa)	Elastic Modulus (GPa)
Sn-20Bi/Cu	6.8841	174.7852
Sn-20Bi-0.02GNS/Cu	3.6355	87.5768
Sn-20Bi-0.04GNS/Cu	1.6060	36.4999
Sn-20Bi-0.06GNS/Cu	3.9644	69.6733
Sn-20Bi-0.08GNS/Cu	7.9628	110.1166
Sn-20Bi-0.1GNS/Cu	6.7830	92.6174

**Table 5:** The ratio of hardness/elastic modulus of IMCs at the Sn-20Bi-xGNS/Cu solder joint.

Solder Joint	H/E
Sn-20Bi/Cu	0.0424
Sn-20Bi-0.02GNS/Cu	0.0415
Sn-20Bi-0.04GNS/Cu	0.0435
Sn-20Bi-0.06GNS/Cu	0.0597
Sn-20Bi-0.08GNS/Cu	0.0799
Sn-20Bi-0.1GNS/Cu	0.0743
